# The anxious-depressive attack severity scale: development and initial validation and reliability

**DOI:** 10.1186/s13030-021-00214-1

**Published:** 2021-07-02

**Authors:** Shota Noda, Satomi Matsumoto, Naoko Kawasaki, Mina Masaki, Itaru Fukui, Hisanobu Kaiya

**Affiliations:** 1 Panic Disorder Research Center, Warakukai Medical Corporation, 3-9-18 Akasaka, Minato-ku, Tokyo, 107-0052 Japan; 2 Tokyo Mindfulness Center, 3-9-18 Akasaka, Minato-ku, Tokyo, 107-0052 Japan; 3 Akasaka Clinic, Warakukai Medical Corporation, 3-9-18 Akasaka, Minato-ku, Tokyo, 107-0052 Japan; 4 Nagoya Mental Clinic, Warakukai Medical Corporation, 1-16, Tsubakicho, Nakamura-ku, Nagoya, Aichi 453-0015 Japan; 5 CBT Center, Warakukai Medical Corporation, 3-9-18 Akasaka, Minato-ku, Tokyo, 107-0052 Japan; 6 Faculty of Humanities, Tokyo Kasei University, 1-18-1 Kaga, Itabashi, Tokyo, 173-8602 Japan

**Keywords:** Anxious-depressive attack severity scale, Anxious-depressive attack, Anxious depression, Reliability, Validity

## Abstract

**Background:**

Anxious-depressive attack (ADA) is a symptom complex that comprises sudden intense feelings of anxiety or depression, intrusive rumination of regretful memories or future worries, emotional distress due to painful thoughts, and coping behaviors to manage emotional distress. ADA has been observed trans-diagnostically across various psychiatric disorders. Although the importance of ADA treatment has been indicated, a scale to measure the severity of ADA has not been developed. This study aimed to develop an Anxious-Depressive Attack Severity Scale (ADAS) to measure the severity of ADA symptoms and examine its reliability and validity.

**Methods:**

A total of 242 outpatients responded to a questionnaire and participated in an interview, which were designed to measure the severity of ADA, depressive, anxiety, anxious depression, and social anxiety symptoms. Based on the diagnostic criteria for ADA, 54 patients were confirmed to have ADA and were included in the main study analyses.

**Results:**

The exploratory factor analysis of the ADAS identified a two factor structure: severity of ADA symptoms and ADA frequency and coping behaviors. McDonald’s ωt coefficients were high for the overall scale and the first factor (ωt = .78 and ωt = .83, respectively) but low for the second factor (ωt = .49). The ADAS score was significantly positively correlated with clinical symptoms related to anxiety and depression.

**Conclusion:**

The present study demonstrated that the ADAS has sufficient reliability and validity; however, internal consistency was insufficient for the second factor. Overall, the ADAS has potential to be a valuable tool for use in clinical trials of ADA.

## Background

Anxious-depressive attack (ADA) is a novel cluster of symptoms that include abrupt outbursts of anxiety or depression, intrusive rumination of negative memories or future worries (with or without flashbacks), intense emotional distress that is caused by recalling painful details of past memories or anticipatory concerns, and a wide range of violent coping behaviors for emotional distress, including self-harm and overdosing [1]. There is no direct psychological cause of ADA, and it is thought to be a psychological form of a panic attack.

Table 1 shows the diagnostic criteria of ADA [2]. ADA differs from panic attacks. It does not include intense physical symptoms such as those observed in panic disorder, and its core symptoms include severe anxiety, intrusive rumination or worry, and emotional distress caused by these thoughts. The attack also exhibits negative feelings such as depression, sadness, self-hatred, emptiness, helplessness, anxious-irritable feelings, and loneliness [1]. Indeed, ADA is a trans-diagnostic symptom complex in patients with various anxiety disorders as well as mood disorders [1, 3]. ADA frequency has been shown to be correlated with the severity of social anxiety and depressive symptoms [1]. In a previous study of patients with social anxiety disorder, the relation between ADA, social anxiety, depressive symptoms, and rejection sensitivity was examined using structural equation modeling [2]. The results showed that ADA was directly affected by rejection sensitivity and depressive symptoms and indirectly affected by social anxiety symptoms via depressive symptoms.

**Table 1:** Diagnostic criteria of anxious-depressive attack [2]

A. Anxious-depressive attack occurs suddenly and recurrently regardless of one’s situation in various mental disorders.	
B. The following symptoms proceed in descending order, but symptom no. 4 is elective.	
1. Abrupt surge of intense discomfort consisting of mixed emotions of anxious and depressive nature with or without being moved to tears. A peak comes within several seconds or less than a minute (sudden intense feelings of anxiety or depression).	
2. Intrusive rumination including mostly negative memories, consisting of mainly recent or past adverse events (flashbacks) or rarely worry, which continues for several tens of minutes to several hours (intrusive rumination of regretful memories or future worries).	
3. Prominent agitation, unrest, or loneliness that occurs during rumination and was very violent and inappropriate to ruminative contents (emotional distress due to painful thoughts).	
4. Various coping behaviors to manage intense discomfort occasionally appear (coping behaviors to manage emotional distress).	
C. Physical symptoms, e.g., shortness of breath and palpitations, are extremely modest.	
D. The disturbance is not attributable to the direct psychological effects of any stress, physiological effects of a substance, or a neurological or other medical condition.	
E. The disturbance is not better explained by another neuropsychiatric disorder (e.g., panic disorder, post-traumatic stress disorder, non-epileptic seizure, frontal epilepsy, intermittent explosive disorder, anxious distress specified for depression, sudden emotional excitement of schizophrenia, or Ataque de nervios).	

The prevalence of ADA in new patients visiting clinics solely for anxiety and mood disorders was estimated as 16.88% [4], which indicates that ADA is not a rare symptom complex. Furthermore, ADA generally has a refractory and chronic nature requiring treatment [1, 3]. Although a questionnaire to confirm the presence of ADA has been developed [1], a scale to measure the severity of ADA symptom has not. Therefore, the present study aimed to develop an Anxious-Depressive Attack Severity Scale (ADAS). We examined the reliability and validity of the ADAS by correlating the scores with severity of depressive, anxiety, anxious depression, and social anxiety symptoms.

## Methods

### Participants

Participants were outpatients who visited clinics solely for anxiety and depression in Tokyo and Yokohama and were aged ≥16 years. Exclusion criteria included high suicide risk, severe physical illness, and significant cognitive impairment. After obtaining written informed consent, 242 outpatients participated in a survey. Of these, 54 patients (10 men and 44 women) were confirmed to have experienced ADA according to the diagnostic criteria of ADA (Table 1). The age of participants ranged from 16 to 78 years, with a mean age of 33.67 (standard deviation [*SD*] = 13.17) years. Table 2 summarizes the clinical characteristics of the participants with ADA according to a Mini-International Neuropsychiatric Interview (MINI: [5, 6]) conducted by clinical psychologists.

**Table 2:** Clinical characteristics of participants with ADA

Characteristic	Number (rate)	Sample size
Male (%)	10 (18.52%)	54
Mean age (SD)	33.67 (13.17)	54
MINI Diagnosis (%)		
Major depressive episode, current	22 (43.14%)	51
Major depressive episode, past	16 (31.37%)	51
Dysthymia	16 (31.37%)	51
Manic episode, current	0	51
Manic episode, past	3 (5.88%)	51
Hypomanic episode, current	1 (1.96%)	51
Hypomanic episode, past	6 (11.76%)	51
Current panic disorder	10 (19.61%)	51
Agoraphobia	17 (33.33%)	51
Social anxiety disorder	12 (23.53%)	51
Obsessive-compulsive disorder	4 (7.84%)	51
Post-traumatic stress disorder	0	51
Alcohol dependence	4 (7.84%)	51
Alcohol abuse	1 (1.96%)	51
Substance dependence	1 (1.96%)	51
Substance abuse	1 (1.96%)	51
Psychotic disorders	0	51
Mood disorder with psychotic features lifetime	1 (1.96%)	51
Anorexia nervosa	0	51
Bulimia nervosa	2 (3.92%)	51
Anorexia nervosa, binge eating/purging type	0	51
Generalized anxiety disorder	17 (33.33%)	51

*MINI* Mini-International Neuropsychiatric Interview

This study was approved by the ethics committee of the first author’s affiliated institution.

### Measures

The ADAS was developed to assess the severity of ADA symptoms. We developed seven items to measure the severity of ADA symptoms. These items included four symptoms (Item 1, Diagnostic Criteria B-1: sudden intense feelings of anxiety or depression; Item 2, Diagnostic Criteria B-2: intrusive rumination of regretful memories or future worries; Item 3, Diagnostic Criteria B-3: emotional distress caused by painful thoughts; and Item 4, Diagnostic Criteria B-4: coping behaviors to manage emotional distress) based on the diagnostic criteria of ADA (Table 1). We also added items focusing on ADA frequency and duration according to the study by Kaiya [1]. We included an item on the overall ADA severity to measure the overall severity of symptoms, including the patient’s subjective pain and impairment in life. The seven items are listed in Table 3. The ADAS was administered using the structured interview method.

**Table 3:** Original items of the Anxious-Depressive Attack Severity Scale

1	Have the sudden unpleasant emotions occurred without any triggering events? How severe are the abrupt emotions in the anxious-depressive attack? (Diagnostic Criteria B-1)
2	Did your past memories automatically come out following the emotional attacks? How do those memories come back to you? Which better reflects your experience; 1. a memory comes back to you slowly or 2. your memories come out one after another? And if you want to stop remembering that memory, can you stop it? (Diagnostic Criteria B-2)
3	Did you have unpleasant emotions while remembering such past events? How severe was the emotional distress? (Diagnostic Criteria B-3)
4	Did you take any action to avoid such painful experiences? (Diagnostic Criteria B-4)
5	How frequent were your anxious-depressive attacks in the last two weeks? (ADA frequency)
6	What was the average duration of your anxious-depressive attacks during the last two weeks? (ADA duration)
7	What was the average severity of the overall anxious-depressive attacks in the last two weeks (considering the subjective pain and impairment in life)? (severity of the overall ADA)

Items 1, 2, 3, and 7 are rated on a 4-point Likert scale (0 = none, 1 = mild, 2 = moderate, and 3 = severe); item 4 is rated on a 5-point Likert scale (0 = none, 1 = coping by oneself, 2 = coping with others, 3 = coping by substance intake or escape behavior, 4 = aggressive behavior, substance dependence, or other); item 5 is rated on a 4-point Likert scale (0 = none, 1 = once or twice a week, 2 = three or four times a week, and 3 = five or more times a week); item 6 is rated on a 4-point Likert scale (0 = none, 1 = within 60 min, 2 = 60 to 180 min, and 3 = 180 min or more). Before the implementation of ADAS, ADA can be explained as follows: some people suddenly feel sad (negative feelings) and then recollect unpleasant memories of the past, leading to pain, despite the lack of a triggering event. The symptoms that are manifested are known as anxious-depressive attack.

During the ADAS interview session, five psychological batteries were also administered. The Hamilton Depression Rating Scale (HAM-D: [7, 8]) consists of 17 items and is one of the most widely used scales for the assessment of depressive symptoms. The scale covers the whole spectrum of depressive symptoms, which includes affective, cognitive, and somatic symptoms. Items are scored from 0 to 4 (absent, mild or trivial, moderate, and severe) or 0 to 2 (absent, slight or doubtful, and clearly present). The total score ranges from 0 to 54, with higher scores representing greater severity of depressive symptoms.

The Hamilton Anxiety Rating Scale (HAM-A: [9, 10]) consists of 14 items and is one of the most widely used scales for assessing anxiety symptoms in research settings. Items are scored from 0 to 4 (not present, mild, moderate, severe, and very severe). The total score ranges from 0 to 56, with higher scores indicating greater severity of anxiety symptoms.

The Quick Inventory of Depressive Symptomatology (QIDS: [11, 12]) measures nine symptom domains of depression. The total score ranges from 0 to 27, with higher scores representing higher severity of depressive symptoms.

The Anxious Depression Scale (ADS: [13]) measures anxious depression symptoms in patients with depressive disorder with atypical features. It is a self-reported measure comprising 20 items and consists of 4 factors: behavioral/emotional symptoms, physical symptoms, aggressive emotions, and nonaggressive emotions. Items are scored from 1 to 4 (not at all, sometimes, mostly, and very much) and the total score ranges from 20 to 80.

The Liebowitz Social Anxiety Scale (LSAS: [14, 15]) was originally developed as a clinician-administered scale to assess the range of social interactions and performance situations feared by patients to help diagnose social anxiety disorder. It was subsequently validated as a self-report inventory comprising 24 items, which are each scored on two 4-point Likert scales for level of fear and frequency avoidance during situations, such as “telephoning in public.” The total score ranges from 0 to 144.

### Statistical analyses

First, an exploratory factor analysis (EFA) using principal component analysis (Promax rotation) was conducted to determine the factor structure of the ADAS. Second, item-total correlation and McDonald’s ωt coefficients for the ADAS were computed to examine reliability. Third, to examine the criteria-related validity of the ADAS, we computed Pearson’s correlation coefficients between the ADAS and the HAM-D, HAM-A, QIDS, ADS, and LSAS. SPSS version 25 (IBM Corp., Armonk, NY, USA) was used to conduct the EFA and correlation analyses. R version 4.0.2 was used to compute McDonald’s ωt coefficients and to conduct a parallel analysis.

## Results

### EFA

The Kaiser–Meyer–Olkin (KMO) measure of sampling adequacy was .639 (KMO value ≥ .60); thus, the data were suitable for factor analysis [16]. Bartlett’s test of sphericity was significant (*p* < .01). The eigenvalues of the first, second, third, fourth, and fifth components were 3.01, 1.31. 1.12, .60, and .54, respectively. Three factors were determined using the Kaiser criterion, two factors by the scree plot, and two factors by the parallel analysis results. Based on these indices, two-factor and three-factor structure were assumed and factor analyses were conducted. The results showed that the three-factor structure had a factor with only one item and the three items had factor loadings greater than .30 for two or more factors. Therefore, the two-factor model was found to be the most interpretable solution. The EFA results showed that the ADAS had a two-factor structure, with five items in the first factor (severity of ADA symptoms) and two items in the second factor (ADA frequency and coping behaviors; Table 4).

**Table 4:** Results of the exploratory factor analysis

		Factor loadings		Mean (SD)	Item-total correlations
		I	II		
**I**	**Severity of ADA symptoms**	*ω*t = .83			
3	Emotional distress against the painful thoughts	.95	−.12	2.22(1.11)	.79**
2	Intrusive rumination of regretful memories or future worries	.94	−.17	2.15(1.12)	.77**
7	Severity of overall ADA	.66	.05	2.33(.75)	.61**
1	Sudden intense feelings regarding anxiety or depression	.61	.26	2.52(.67)	.64**
6	Duration of ADA	.51	.27	1.67(.78)	.62**
**II**	**ADA frequency and coping behaviors**	*ω*t = .49			
5	ADA frequency	.01	.80	1.91(.85)	.45**
4	Coping behaviors to manage the emotional distress	−.01	.79	1.69(1.48)	.55**

**p < .01

### Item-total correlations and internal consistency

The results of the item-total correlation analyses showed that there were moderate to strong positive correlations between the total score and each item of the ADAS (*r* = .45–.79, *p* < .01) (Table 4). Furthermore, McDonald’s ωt coefficients were high for the overall scale and first factor (ωt = .78, ωt = .83) and low for the second factor (ωt = .49).

### Criterion-related validity

The ADAS total score was significantly and positively correlated with HAM-D, HAM-A, QIDS, ADS, and LSAS scores (*p* < .05; Table 5). The first factor of the ADAS showed significant and positive correlations with HAM-D, QIDS, ADS, and LSAS scores (*p* < .05), but not with the HAM-A score. The second factor of the ADAS showed significant positive correlations with HAM-D and HAM-A scores (*p* < .05), but not with QIDS, ADS, and LSAS scores.

**Table 5:** Correlations between the ADAS and other measures

		Mean	SD	HAM-D	HAM-A	QIDS	ADS	LSAS
				(*N* = 53)	(*N* = 52)	(*N* = 54)	(*N* = 50)	(*N* = 53)
1	ADAS	14.48	4.34	.43**	.27*	.38**	.47**	.27*
2	Severity of ADA symptoms	10.89	3.49	.34**	.17	.35**	.48**	.33**
3	ADA frequency and coping behaviors	3.59	1.93	.33**	.29*	.22	.20	.02

*ADAS* Anxious Depression Attack Severity Scale, *HAM-D* Hamilton Depression Rating Scale, *HAM-A* Hamilton Anxiety Rating Scale, *QIDS* Quick Inventory of Depression Symptomatology, *ADS* Anxious Depression Scale, *LSAS* Liebowitz Social Anxiety Scale

**p < .01, *p < .05

## Discussion

The goal of the present study was to develop the ADAS and examine its reliability and validity. The EFA showed that the ADAS had a two-factor structure: “severity of ADA symptoms” factor (five items) and “ADA frequency and coping behaviors” factor (two items). The severity of ADA symptom (the first factor) was strongly related to the intrusive rumination of regretful memories or future worries and the emotional distress caused by painful thoughts. The ADA frequency and coping behaviors (the second factor) consisted of ADA frequency and coping behaviors to manage emotional distress. Furthermore, the correlation coefficients between each item and the total score ranged from .45 to .79. McDonald’s ωt coefficients of the ADAS for the overall scale and first factor were higher than .75, which indicated high internal consistency. However, the ωt coefficient for the second factor was low. There were only two items in the second factor, which may have contributed to the low ωt coefficient.

The criterion-related validity of the ADAS was assessed by examining whether or not the ADAS scores correlated with clinical indices that are associated with ADA. The ADAS showed significant positive correlations with the severity of depressive, anxiety, anxious depression, and social anxiety symptoms, and these results are similar to those observed in previous studies [1–3]. Hence, the ADAS has criterion-related validity. These findings suggested that the ADAS is a reliable and valid tool for assessing the severity of ADA.

As shown in Table 2, ADA is predominantly observed in patients with depression and/or anxiety disorders. ADA was identified in 43.14, 33.33, 23.53, and 19.61% of patients with major depressive episodes, agoraphobia and generalized anxiety disorder, social anxiety disorder, and current panic disorder, respectively. Therefore, ADA is a trans-diagnostic symptom complex, particularly those occurring in anxiety and depressive disorders.

Previous studies have highlighted the importance of treating ADA and the need for ADA assessment tool [1, 3]. The ADAS findings in this study showed that many patients had moderate-to-severe ADA symptoms. In item 7, which assesses the severity of overall ADA, the mean score was 2.33: total score 3, and 27 of 54 patients fell into the severe category (score 3). Thus, many patients suffer from ADA symptoms and require treatment. ADAS will enable the accurate assessment of the degree of ADA symptoms and could be a useful screening tool for patients requiring ADA treatment. ADAS is also expected to contribute to the understanding of the ADA pathology. Future studies are required to examine the relation between the severity of ADA symptoms as measured by ADAS and psychological, physiological, and social indicators.

However, there are some limitations to this study that need to be considered. The internal consistency for the second factor (ADA frequency and coping behaviors factor) was low. The number of items in the second factor should be increased to improve the internal consistency. On achieving improvement, a priori power calculations should be performed and then cross-validity should be assessed in a larger sample of patients for more reliable results. A longitudinal study that evaluates ADAS sensitivity to change would also be useful.

## Conclusions

In the present study, our newly developed ADAS was shown to be a reliable and valid instrument for assessing the severity of ADA. The ADAS can be a valuable tool for use in clinical trials of ADA.

## Data Availability

Detailed data are available from the corresponding authors upon reasonable request.

## Abbreviations

ADA: Anxious-depressive attack
ADAS: Anxious-Depressive Attack Severity Scale
HAM-D: Hamilton depression rating scale
HAM-A: Hamilton anxiety rating scale
QIDS: Quick inventory of depressive symptomatology
ADS: Anxious depression scale
LSAS: Liebowitz social anxiety scale
SD: Standard deviation
